# ZnO Nanowires/Self-Assembled Monolayer Mediated Selective Detection of Hydrogen

**DOI:** 10.3390/s24217011

**Published:** 2024-10-31

**Authors:** Mandeep Singh, Navpreet Kaur, Elisabetta Comini

**Affiliations:** 1 Physics Department, Politecnico di Milano, Piazza L. da Vinci 32, 20133 Milano, Italy; 2 SENSOR Laboratory, University of Brescia, Via D. Valotti 9, 25133 Brescia, Italy

**Keywords:** gas sensors, metal oxide, nanowires, self-assembled monolayer, hydrogen

## Abstract

We are proposing a novel self-assembled monolayer (SAM) functionalized ZnO nanowires (NWs)-based conductometric sensor for the selective detection of hydrogen (H_2_). The modulation of the surface electron density of ZnO NWs due to the presence of negatively charged terminal amine groups (−NH_2_) of monolayers leads to an enhanced electron donation from H_2_ to ZnO NWs. This, in turn, increases the relative change in the conductance (response) of functionalized ZnO NWs as compared to bare ones. In contrast, the sensing mechanism of bare ZnO NWs is determined by the chemisorbed oxygen ions. The functionalized ZnO NWs exhibit an eight times higher response compared to bare ZnO NWs at an optimal working temperature of 200 °C. Finally, in comparison to studies in the literature involving strategies to enhance the sensing performance of metal oxides toward H_2_, like decoration with metal nanoparticles, heterostructures, and functionalization with a metal–organic framework, etc., SAM functionalization showed superior sensing results.

## 1. Introduction

Self-assembled monolayers (SAMs) are two-dimensional (2D) highly ordered molecular arrangements that can be formed spontaneously on a variety of surfaces, including metal oxides (MOXs) [1,2,3]. The typical structure of SAMs contains three units: the head group (binds to the surface), the backbone (provides the molecular ordering), and the end or terminal group (defines the functionality of the surface) [1]. As the terminal group of SAMs defines the functionality, it can further be used for various purposes, such as the immobilization and detection of biomolecules [1]. Therefore, the functionalized surfaces of SAMs have been extensively used for biosensing applications [4,5,6]. Another area in which SAM functionalization was found to be extremely useful is in improving the charge carrier injection at the electrode/semiconductor interface via tuning the work function of an electrode [3,7,8]. Indeed, the performance of devices such as organic thin film transistors [8,9], light emitting diodes [10,11], solar cells [12,13], etc., were found to improve after tuning the work function of electrodes. In recent years, SAM-functionalized MOXs such as ZnO [14,15], WO_3_ [16], and NiO [17], etc., in nanowires (NWs) form have been used for the detection of gaseous compounds. MOXs NWs-based gas sensors exhibit fast response/recovery dynamics, single crystallinity, excellent charge transport properties, and lower detection limits that will help to develop robust, ultrasensitive, and stable sensing devices [18]. The first significant work was conducted by Hoffman et al. [19], in which SnO_2_ nanowires (NWs) were functionalized with [3-(trimethoxysilyl)propyl]ethylenediamine) and explored for the selective detection of NO_2_ (oxidizing gas). In another work, (3-aminopropyl)trimethoxysilane (APTES)-functionalized WO_3_ nanotubes were used for the selective detection of NO_2_ [16]. On the other hand, our group was focused on the selective detection of reducing volatile organic compounds (VOCs) like acetone via functionalizing ZnO and NiO NWs with organosilanes [14,15]. We have observed that mainly two notable factors were found to improve sensor response and selectivity after SAM functionalization: (i) unique molecular interactions between the terminal groups of SAMs and gas analytes, and (ii) the modulation of surface carrier density by polar SAM terminal groups. Another reducing compound whose detection at the current time is of utmost importance is hydrogen (H_2_) [20,21]. Along with solar energy, H_2_ is seen as a future fuel because of its high abundance, efficiency, renewability, and eco-friendliness. Consequently, an immense amount of research has been focused on the use of hydrogen as a fuel [22] and its production [23,24]. However, because of its highly flammable nature [25], ensuring the safe and controlled production, storage, and utilization of hydrogen is paramount. This requires an ultrasensitive, real-time monitoring and sensing platform for the selective detection of H_2_.

Herein, we are presenting a novel APTES-functionalized ZnO NWs-based conductometric sensor for the selective detection of H_2_. According to the best of our knowledge, no report is available in the literature in which SAMs-functionalized MOXs were explored for H_2_ detection. We show that via modulating the surface electron density with the polar SAM end groups, i.e., negatively charged −NH_2_ groups of APTES, the sensitivity, response, and selectivity of ZnO NWs sensors can be tuned. The ZnO NWs were synthesized using a vapor–liquid–solid mechanism and afterwards functionalized with organosilanes via the simple dip method. After systematic characterization, sets of conductometric sensors of bare and SAMs-functionalized NWs were fabricated using magnetron sputtering and, finally, were explored for the detection of H_2_. We also compared the performance of our sensors with comprehensive cases from the literature involving different strategies employed to detect hydrogen and showed that the SAMs functionalization of metal oxides is a superior approach. We would like to point out that the detailed and deep surface chemical properties of APTES monolayer-functionalized ZnO NWs were already investigated in our previous work [26], while this article is focused on H_2_ sensing.

## 2. Materials and Methods

### 2.1. Substrate Preparation, Deposition of Gold Catalyst, and Growth of ZnO Nanowires

Alumina substrates measuring 3 × 3 mm^2^ (Al_2_O_3_, 99% purity, Kyocera, Japan) were used to grow the ZnO NWs. Before the synthesis of the NWs, the substrates were cleaned ultrasonically with acetone and dried using synthetic air. Then, the deposition of gold (Au) catalyst on Al_2_O_3_ substrates using RF magnetron sputtering was conducted. The deposition was carried out at 7 SCCM Ar flow for 5 s using an electrical power of 70 W at a pressure of 5 × 10^−3^ mbar.

VLS mechanisms were used for the growth of the ZnO NWs, and it was carried out in a tubular furnace (a custom design based on a commercial Lenton furnace). The source material, i.e., the ZnO powder (Sigma-Aldrich, Burlington, MA, USA, 99% purity) in an alumina crucible, was placed in the middle of the furnace, which has a higher temperature region, while the Au-catalyzed substrates were placed at a relatively lower temperature region to promote the condensation of evaporated material. The carrier gas (argon, Ar) was used to transport the vapors of the source material to the substrates. The pressure inside the alumina tube was maintained at 10 mbar. In this work, the synthesis of the ZnO NWs was conducted at 1200 °C while the substrates were kept at 380 °C. During the growth of the NWs, the argon (carrier gas) flow was set at 100 SCCM with the deposition time set to 12 min. In the first stage, the furnace temperature was raised to 1200 °C, and the flow of argon was set from the substrates to the source material’s direction to avoid any undesirable deposition of ZnO powder on the substrate. When the temperature reached deposition temperature, the argon flow was directed from the source to the substrate. The VLS mechanism is named after the three different phases of the material: the vapor phase of the source material (ZnO powder), the liquid phase of the catalyst droplet (Au) at a given substrate temperature, and the solid crystalline nanostructure formed during the process. Hence, during the ZnO NWs synthesis, the ZnO vapor adsorption on the liquid droplet of the Au catalyst occurs at 380 °C. As the vapors of the ZnO are continuously provided for 12 min, the liquid alloy starts to saturate, forming a solid precipitate that grows in the form of a 1D nanostructure.

### 2.2. Surface Functionalization of ZnO Nanowires with APTES Monolayer

The ZnO NWs were functionalized with APTES self-assembled monolayers using a simple dip method. APTES is an organosilane that requires hydroxyl groups (—OH) for its attachment to ZnO surfaces. To form —OH groups, surface treatment such as with piranha solution [1] or strong hydroxides (e.g., NaOH, KOH) [17] can be used. However, a ZnO surface is usually enriched with —OH groups, thus requiring no such kind of surface treatment [15]. For functionalization, a 10 mM solution of APTES in absolute ethanol was prepared. The ZnO NWs substrates were dipped inside each solution for 18 h at room temperature under a smooth stirring. While undergoing silanization, the APTES molecules covalently bound to the ZnO surface by forming polysiloxane bonds, establishing connections with the surface silanol groups (–SiOH) through Si-O-Si bonds. Indeed, the process of self-assembled monolayer (SAM) attachment to the surface can be delineated into two distinct stages. Initially, a thin monolayer forms rapidly through the adsorption of SAM molecules from the bulk solution, typically completing within a few minutes. Subsequently, a slow rearrangement and reorientation of the adsorbed SAM molecules takes place over a prolonged period (typically 10–20 h) to achieve a highly ordered structure. Once removed from the solution, the SAMs-modified ZnO samples underwent extensive rinsing with ethanol and drying with synthetic air to eliminate physically adsorbed APTES molecules. Subsequently, the samples were heated to 80 °C to ensure complete removal of solvent residue. As the terminal or end group of APTES is amine (−NH_2_), amine-terminated ZnO NWs are obtained after functionalization. For the confirmation of surface functionalization and the surface chemical properties of the APTES monolayer, X-ray photoelectron spectroscopy (XPS) was performed and is reported in our previous article [26].

### 2.3. Conductometric Gas Sensor Device Fabrications and Measurements

**Figure 1:**
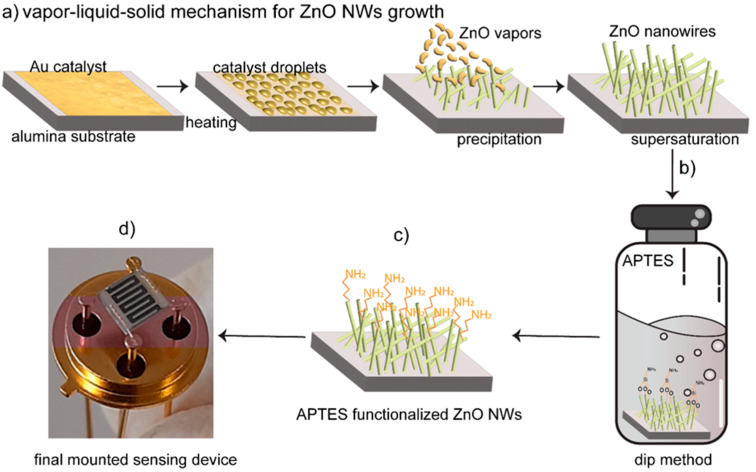
(**a**) Steps involved in the growth of ZnO NWs using a VLS mechanism, (**b**) functionalization of ZnO NWs with an APTES monolayer using the dip method, (**c**) amine-terminated ZnO NWs, (**d**) a TO package mounted conductometric sensor.

The conductometric sensing devices of bare and APTES-functionalized ZnO NWs devices were fabricated using DC magnetron sputtering. The complete sensor integration process involved the following: the deposition of a 50 nm titanium–tungsten alloy (TiW) adhesion layer (70 W argon plasma, RT, and pressure (5.3 × 10^−3^ mbar), 3 min) on top of ZnO NWs, which is followed by a 1 μm platinum interdigitate contact (20 min) employing the same deposition conditions. On the back side of the alumina substrates, a heating element was deposited following the above-mentioned conditions. Finally, the fabricated devices were mounted on TO packages using electro-soldered gold wires. The APTES-functionalized sensors were denoted as Z_APTES. The whole experimental process, i.e., the deposition of the Au catalyst on the alumina substrate, the heating of the substrate inside the furnace, the formation of Au droplets, the deposition of ZnO vapors on the Au droplets containing the substrates, the growth of nanowires, the surface functionalization of the ZnO NWs with the dip method, and finally the fabrication of the conductometric sensor, is depicted in Figure 1.

The conductometric response of the sensors was investigated using a flow-through technique. A custom stainless-steel chamber (1 L volume) located inside a climatic chamber (Angelantoni, Italy, model MTC 120) set at 20 °C was used to investigate the sensor response toward different gases. Before the sensing measurements, the thermal stabilization of the sensors at the desired working temperature was conducted for 6 h. Different types of oxidizing and reducing gases and their concentrations were used, and the electrical conductance of the sensors was recorded. Each target gas was mixed with synthetic air (SIAD, Bergamo, Italy), generating a total flow inside the chamber of 200 SCCM within dry air at 20 °C. The voltage applied to the sensor electrodes was fixed at 1 V. For ZnO NWs, which are an n-type metal oxide, the response is determined by the variation of conductance using the following formulas for reducing gas: [14]

(1) $$Response=\left(G_{gas}-G_{air}\right)∕G_{air}$$

and for oxidizing gas:

(2) $$Response=\left(G_{air}-G_{gas}\right)∕G_{gas}$$

where the G_gas_ and G_air_ are the sensor conductance in gas and synthetic air, respectively. Specifically, different concentrations of hydrogen gas (50, 200, 300, 500 ppm) at different temperatures (200, 300, 400, 500 °C) were tested. Other interfering reducing and oxidizing gas analytes, such as ethanol, acetone, nitrogen dioxide, and carbon monoxide, were tested during the sensing measurements. Further, the experimental data from the calibration curves were fitted by typical power trend relations for metal oxide sensors [27,28].

(3) $$Response=A{\left[Concentration\right]}^{B}$$

where A and B are constants typical of the sensor material and stoichiometry of the involved reaction. The detection limits for the H_2_ gas were calculated by considering a minimum response of 1 as having a detectable signal.

## 3. Results

### 3.1. Surface Morphology and Crystallinity of Nanowires

**Figure 2:**
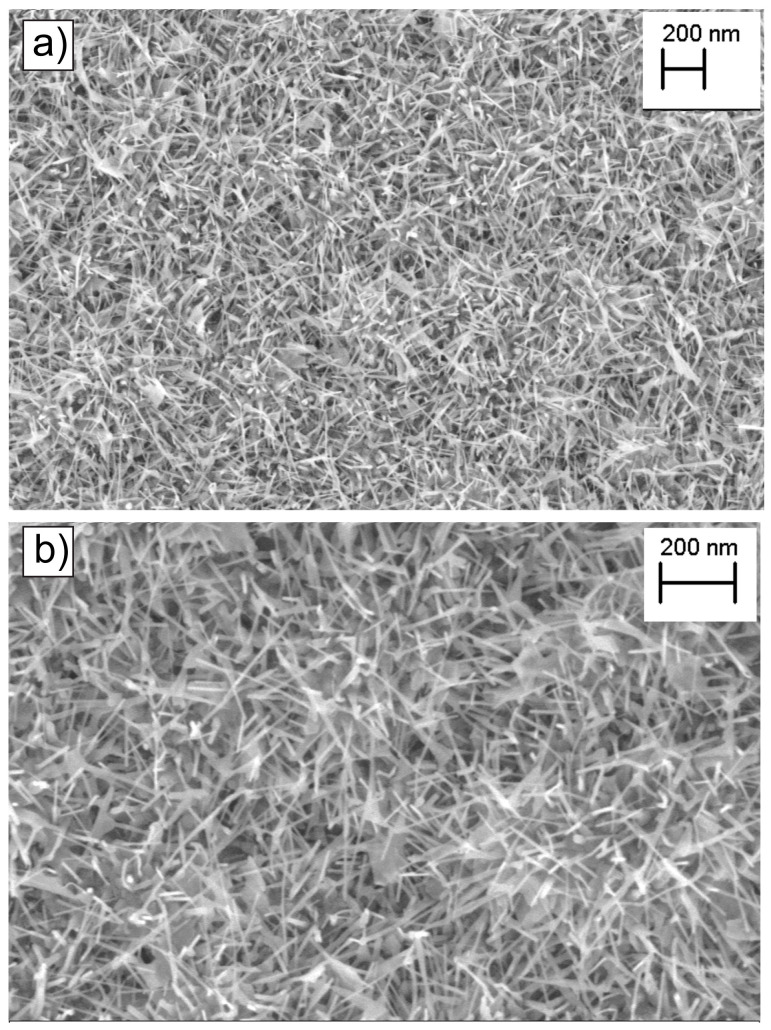
SEM images of ZnO nanowires at lower (**a**) and higher (**b**), magnifications.

The surface morphology of pristine ZnO NWs is shown in Figure 2a,b. Specifically, SEM images of the ZnO NWs were shown at different magnifications. The grown NWs exhibit dense and uniform morphology, covering the entire Al_2_O_3_ substrate with a random orientation of growth. The average diameter of the NWs was found to be 35 ± 9 nm, while the length was found to be on a micrometer scale. Furthermore, the GI-XRD spectra of the ZnO NWs were also presented in Figure S1. Detailed analysis showed that the ZnO NWs were polycrystalline in nature with a hexagonal wurtzite crystal structure [29].

### 3.2. Surface Chemical Composition of APTES-Functionalized ZnO NWs

As mentioned earlier, the detailed analysis of the APTES monolayer was presented in our previous report. Briefly, in the case of functionalized ZnO NWs, two distinct peaks, i.e., N1S and Si2p, were observed, which belong to the APTES monolayer (absent in the case of the bare ZnO NWs). The Si2p peak was centered around BE = 105 eV. Meanwhile, the N1S peak contained two contributions: one at BE = 399.6 eV, which belonged to the primary amine, and the second one at BE = 398.2 eV, attributed to the formation of Si-N bridges in the self-assembly process. These observations confirmed the successful formation of —NH_2_-terminated ZnO NWs.

### 3.3. Sensing Performance of Bare and APTES-Functionalized ZnO Nanowires

Before discussing the results, we would like to point out that we first scanned the sensor at different temperatures towards the different concentrations of gas analyte to find the optimal working conditions. However, we have chosen the following representation of the sensing data according to the focus of our work.

**Figure 3:**
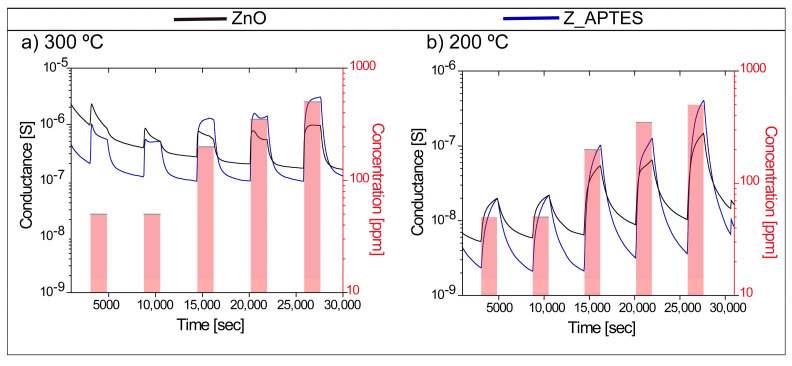
Dynamic response of bare and APTES-functionalized ZnO NWs at (**a**) 300 °C and (**b**) 200 °C.

Figure 3a,b reports the dynamic transient response of bare and APTES-functionalized ZnO NWs (Z_APTES) sensors at 300 °C (Figure 3a) and 200 °C (Figure 3b) in relation to different concentrations of hydrogen (50, 50, 200, 300, 500 ppm) in dry air. Both types of sensors show an increase in electrical conductance after reacting with H_2_ (reducing gas). This is the typical behavior of an n-type metal oxide sensor upon reacting with a reducing compound (electron donating gas) [15]. Clearly, the relative change in the conductance after reaction with H_2_ is much higher at 200 °C than 300 °C, attributed to the superior sensing performance at 200 °C in relation to H_2_. However, the response and recovery of the dynamics at 300 °C are faster than for both the ZnO and functionalized sensors. A possible reason for slow recovery at 200 °C is associated with low thermal excitation, i.e., poor charge transport at 200 °C as compared to 300 °C. The calculated values of the response and recovery times for the bare and APTES-functionalized ZnO NWs were calculated and are shown in Figure S2 (see SI). The response time is the time required for a sensor resistance/conductance to reach 90% of the equilibrium value under the exposure of a gas analyte, while the recovery time is the time required for a sensor resistance/conductance to return to 70% of its original value of resistance/conductance in air [30].

**Figure 4:**
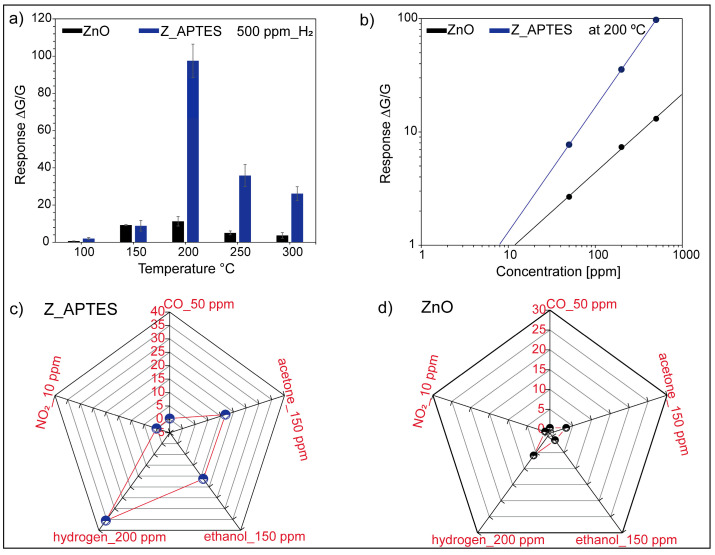
(**a**) Response vs. temperature graph of bare and APTES-functionalized ZnO NWs toward 500 ppm of H_2_, (**b**) calibration curves of bare and APTES-functionalized ZnO NWs at 200 °C, (**c**,**d**) response of bare and APTES-functionalized ZnO NWs toward different gas analytes at the optimal working temperature.

Furthermore, the sensors were operated at different temperatures (100, 150, 200, 250, and 300 °C) and their response toward 500 ppm of H_2_ is shown in Figure 4a. In order to determine the sensor performance reproducibility, the performance of three different sensors was tested, and their average response with error bars is presented in Figure 4a. Temperature is pivotal in metal oxide-based sensors as it alters the composition of adsorbed oxygen ions on their surfaces [30]. These ions dictate the sensor’s response to a particular gas analyte, thereby offering a means to adjust their selectivity. At lower temperatures (100–150 °C), the sensors exhibit low response, which is attributed to the poor thermally induced charge transport in both bare and functionalized ZnO NWs. As the temperature increased, the charge carriers gained enough thermal energy, and the sensors started showing better sensing performance. As can be seen in Figure 4a, all sensors exhibited the highest response value at 200 °C, indicating it as an optimal working temperature. The response of the sensors seemed to decrease with the further increase in temperature beyond 200 °C. Indeed, under optimal conditions, the response of the Z_APTES sensors exhibited superior performance, with response values approximately eight times higher than the bare ZnO NWs. In our last work [16], we found that at 300 °C, APTES-functionalized ZnO NWs showed selective sensing performance toward acetone. This means that, via changing the temperature, the selectivity of Z_APTES sensors can be tuned. Hence, APTES-functionalized ZnO NWs offer a unique possibility to develop different sensors just by changing the operating temperature. Furthermore, in Figure 4b, the calibration curves of the bare and Z_APTES sensors at 200 °C that were fitted with Equation (5) are shown. Clearly, the slopes of both curves are different, indicating the different reaction mechanisms of both the sensors. In the bare ZnO NWs, chemisorbed oxygen ions determined their sensing performance, while after functionalization, the modulation of surface electron density via negatively charged −NH_2_ groups of APTES plays the key role behind their distinct and superior sensing behavior. The values of the detection limits and constants (A and B) for the bare ZnO NWs and Z_APTES are presented in Table S1. Indeed, the detection limits of the bare ZnO NWs and Z_APTES were found to be 12 ppm and 7 ppm, respectively. Hence, APTES functionalization not only enhanced the response but also improved the detection limit of ZnO NWs. Furthermore, to investigate selectivity at the optimal working temperature (200 °C), the sensors were exposed to different gases (acetone 150 ppm, ethanol 150 ppm, carbon monoxide 50 ppm, nitrogen dioxide 10 ppm, and hydrogen 200 ppm). The reason behind choosing different concentration values for each of the gases is to be in accordance with their exposure limits. As an example, testing a high concentration of NO_2_, such as 50 ppm, has no significance in real testing conditions as the average exposure limit to NO_2_ must be lower than 0.2 ppm over a period of 1 h, according to the European Union (EU) Air Quality Standards [31]. As seen in Figure 4c,d, the Z_APTES sensors showed a higher selective response toward H_2_ compared to other interfering gases. This behavior mirrors that of the ZnO sensor illustrated in Figure 4d. Indeed, when comparing the response and selectivity of functionalized sensors with bare ZnO NWs, it was seen that the Z_APTES sensors exhibited the highest response and selectivity response toward H_2_ at 200 °C.

Additionally, the effect of the molar ratio on the sensing performance of APTES-functionalized sensors has also been investigated and is presented in Section 4 of the SM. For this, ZnO NWs were functionalized with a twice molar concentration of APTES (20 mM) and their sensing performance was determined and compared with the sensors functionalized with 10 mM of APTES. The reason behind increasing the molar concentration was to enhance the effect of the negatively charged terminal −NH_2_ on the surface electron density of the ZnO NWs, in order to further improve the sensing performance in relation to H_2_. However, exactly the opposite behavior was observed (see Figure S3), and the detailed comparison is presented in the SM. Briefly, the incomplete hydrolysis of APTES molecules inside the SAM solution hindered the uniformity and reactivity of the monolayer [32] that led to a decrease in sensing performance.

### 3.4. Sensing Mechanism

**Figure 5:**
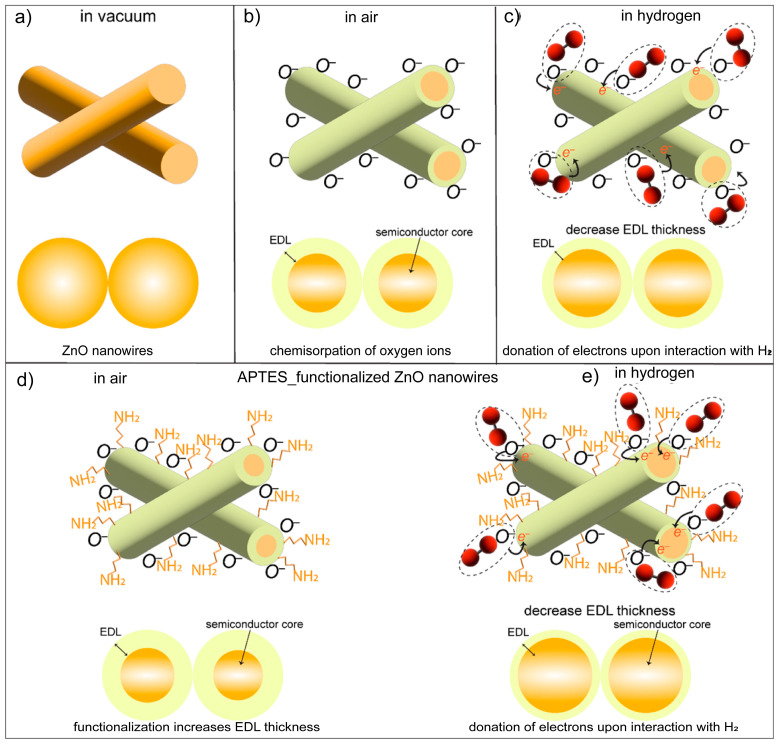
(**a**) ZnO NWs in a vacuum. (**b**) Chemisorption of O¯ on ZnO NWs at 200 °C that led to the formation of a core–shell structure. EDL is the electron depletion layer that forms around the conducting core of the ZnO NWs. (**c**) Interaction of H_2_ with bare ZnO NWs in which electron donation from H_2_ to the ZnO NWs occurs, which in turn increases their conductance. (**d**) Covalent attachment of the APTES monolayer in which negatively charged amine groups push the electrons away from the surface, thus increasing the EDL width. (**e**) Interaction of H_2_ with APTES-functionalized ZnO NWs. The enhanced donation of electrons from H_2_ toward functionalized ZnO NWs enhances their sensing performance as compared to bare ZnO NWs.

ZnO is an intrinsic n-type semiconducting material due to the presence of equilibrium oxygen (anion) vacancies [14,33]. Hence, the majority of charge carriers in ZnO NWs are electrons. When MOXs (ZnO NWs) are operated in air, the chemisorption of oxygen ions occurs on their surface [30]. These chemisorbed oxygen ions extract electrons from their surface and reduce the conductivity of n-type MOXs [30]. This leads to the formation of an electron depletion layer (EDL) shell around the conducting core of n-type MOXs (forms a core–shell type structure) [30]. Figure 5a shows the nanowires in a vacuum, while the chemisorption of oxygen that led to the formation of the core–shell structure when the NWs were operated in air at 200 °C is shown in Figure 5b. In temperature ranging from 100 to 300 °C, the type of chemisorbed oxygen ions on metal oxides is O¯, as shown in the following equation [30]:

(4) $$O_{2}^{-}(ads)+e^{-}↔2O^{-}(ads)$$

**Figure 6:**
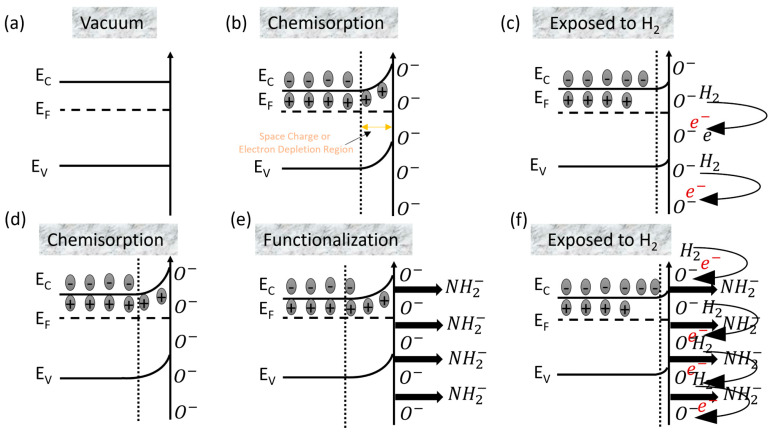
Band bending in bare and APTES-functionalized ZnO nanowires. (**a**) ZnO NWs in vacuum (flat-band). (**b**) chemisorption of O^−^ ions on ZnO NWs that creates electron depletion region (EDL) and band bends upward. (**c**) donation of electronby H_2_ to ZnO NWs decreases the band bending and width of EDL. (**d**) chemisorption of O^−^ ions on APTES functionalized ZnO NWs causes the upward band-bending. (**e**) presence of negatively charged −NH_2_ groups on ZnO NWs pushes the electrons away from surface, thus increases band bending and the width of EDL. (**f**) H_2_ donates electrons to APTES functionalized ZnO NWs and it decreases the band-bending and EDL width.

Equation (4) shows that when MOXs operated between 100 and 300 °C in air, $O_{2}^{-}(ads)$ ions further captured electrons from their surface and became $O^{-}\left(ads\right).$ This can also be explained in terms of the band-bending phenomenon in n-type MOXs. In the first stage, when n-type ZnO NWs are placed in a vacuum, no interaction with the outside environment occurs and the bands are flat (Figure 6a). In the second stage, when NWs are exposed to air, the chemisorption of O^−^ occurs on their surface, which results in the extraction of electrons from the conduction band of ZnO NWs (oxygen trapped electrons). This also causes the bands to bend in an upward direction and creates an electron depletion region, which is also known as a space charge region (Figure 6b). The overall effect of the chemisorption of oxygen ions is a decrease in the conductance of ZnO NWs. Under these conditions, when ZnO NWs are exposed to a reducing gas like H_2_, it donates electrons back to the ZnO NWs which results in a decrease in EDL width and the resistance of the sensor (an increase in conductance as shown in the dynamic response of ZnO NWs, Figure 2). The reaction is pictorially depicted in Figure 5c. In terms of the band bending analogy, the donation of electrons from H_2_ to the ZnO NWs reduces the band bending effect, hence decreasing the space charge electron depletion region width (Figure 6c).

The reaction between H_2_ and the chemisorbed oxygen ions on ZnO NWs can be expressed by the following equation [34]:

(5) $$H_{2}+O^{-}(ads)\to H_{2}O+e^{-}$$

This equation indicates that upon the reaction of bare ZnO NWs with H_2_ gas, H_2_O as a product is also formed in addition to the donation of electrons to the ZnO NWs by H_2_.

In order to understand the sensing mechanism and the reason behind the superior performance of SAMs-functionalized sensors, it is important to address the effect of negatively charged terminal groups, i.e., the effect of the amine of the APTES monolayer on the surface electron density of ZnO NWs and the covalent attachment of APTES with ZnO NWs surfaces. It can be clearly seen from the dynamic response curves (Figure 3) that the baseline conductance of APTES-functionalized ZnO NWs sensors is lower than that of bare ones, which is linked directly to the SAM formation. The first reason behind their lower conductance is the covalent attachment of APTES, in which an electron is extracted from the ZnO NWs’ conduction band, hence reducing their conductance [26]. The second and most important reason is the presence of negatively charged terminal groups of APTES on the n-type ZnO NWs. It has been seen that SAMs with negative or positively changed terminal groups tend to repel or accumulate the surface electron density of n-type semiconductors, respectively [35]. Due to this, the SAM functionalization was markedly employed to improve the performance of organic field effect transistors (OFET) via modulating the charge carrier injections at semiconductor/electrode interfaces [36,37,38]. Specifically, the presence of negatively charged amine terminal groups on the surface generates built-in potential and reduces the surface electron density (it pushes electrons away from the surface) [14]. In terms of band-bending theory, when chemisorbed ZnO NWs were functionalized with the APTES monolayer, the negatively charged terminal —NH_2_groups further pushed the electrons away from the surface, increasing the band banding (Figure 6e). Similar effects of band bending in GaN were observed when functionalized with the self-assembly of phosphonic acid with different electronegativities [39]. This resulted in a further decrease in ZnO NWs conductance and also increased the EDL width (see Figure 4d) [14,15,17]. The decrease in surface electron density and increase in EDL layer width makes SAMs-functionalized ZnO NWs surfaces more favorable for reaction with an electron-donating gas like H_2_ as compared to bare ZnO NWs. Hence, when SAM-functionalized ZnO NWs are exposed to H_2_, they donate more electrons as compared to bare ZnO NWs, and this increases the conductance of functionalized sensors. Indeed, due to this donation of electrons, the width of the EDL layer reduces after the reaction with H_2_ (see Figure 5e and Figure 6f). As the donation of electrons from H_2_ to SAMs-functionalized sensors is higher as compared to bare ZnO NWs, the relative change in conductance (response) is also higher. Hence, both the effect of the negatively charged end groups of SAMs and their covalent attachment with ZnO NWs are the main reasons behind the superior sensing performance of APTES-functionalized sensors at 200 °C.

Now another question arises: why have APTES-functionalized NWs not shown a similar performance to other reducing gases like acetone, ethanol, and CO? The reason behind their selective response only to H_2_ at 200 °C is their different molecular sizes and reaction dynamics. Hydrogen is the smallest and most mobile of the tested reducing gases (i.e., acetone, ethanol, CO). The smaller molecular size of H_2_ allows it to diffuse more efficiently through the APTES-modified layer, facilitating a quicker and stronger interaction with the ZnO surface even at a relatively low temperature. This leads to enhanced sensor response compared to larger, bulkier gas molecules like acetone or ethanol, which may experience diffusion limitations through the functionalized layer.

## 4. Comparison with the Literature

In Table 1, we have compared the performance of the APTES-functionalized ZnO NWs hydrogen sensor with recent reports. For comparison, we have focused on the reports that employed different strategies to enhance the H_2_ sensing performance of metal oxides such as core–shell nanoparticles [40], decoration with metal nanoparticles [40], heterostructures [41], functionalization with a metal–organic framework [42], and so on. Evidently, APTES-functionalized ZnO NWs exhibit the highest response value toward 500 ppm of H_2_ as compared to all other hydrogen sensors tabulated in Table 1. Particularly, the ZIF-8/Pd/ZnO sensor [42] was operated at the same working temperature (200 °C) and showed a response value of 6.7 toward 50 ppm of H_2_, which is lower than the response value of APTES-functionalized ZnO NWs. Furthermore, among all the studies shown in Table 1, ZnO/SnO_2_ nanofiber [43] showed the highest response value of 90; however, the operating temperature was much higher as compared to our sensor. In fact, at 200 °C, our sensors showed a response value of 97 toward 500 ppm of H_2_. Another important strategy to improve the sensing performance of metal oxides that has gathered considerable attention is the use of graphene oxide. We have included three reports in Table 1, in which graphene oxide (GO) and reduced graphene oxide (rGO) [44,45] have been used and clearly APTES-functionalized ZnO NWs exhibit superior sensing performance. Hence, functionalization with SAM is a superior strategy to fabricate highly sensitive and selective H_2_ sensors for hydrogen safety and detection.

## 5. Conclusions

In conclusion, the ZnO NWs were fabricated using a high-yield VLS mechanism and functionalized with an APTES monolayer for the selective detection of H_2_. The ZnO NWs were found to possess hexagonal wurtzite polycrystalline structures with an average diameter of 35 ± 9 nm. The surface functionalization was conducted using the simple dip method, and amine (−NH_2_)-terminated ZnO NWs sensors (Z_APTES) were fabricated. For both the sensors, i.e., Z_APTES and bare ZnO NWs, 200 °C was found to be the optimal working temperature. The Z_APTES showed a superior selective sensing performance toward H_2_, with an eight times higher response value compared to the bare ZnO NWs. Specifically, the Z_APTES exhibited response values of 7.7, 35.5, and 97.5 for 50, 200, and 500 ppm of H_2_. In the case of the bare ZnO NWs, the interaction between H_2_ and chemisorbed oxygen ions was found to determine their sensing mechanism. Meanwhile, the modulation of the surface electron density of the ZnO NWs by a negatively charged terminal −NH_2_ group of APTES monolayers turned out to be the additional and major factor behind their superior sensing performance. Furthermore, the increased molar concentration of SAMs resulted in the deterioration of sensing performance due to the occurrence of incomplete hydrolysis at higher molar concentrations and the formation of disordered monolayers. We have also compared the results with other studies in the literature involving different strategies such as functionalization with a metal–organic framework, graphene, heterostructures, metal particle decoration, etc. We have found that SAM functionalization is a superior strategy to develop an ultrasensitive and selective sensor platform for the detection of H_2_. We believe these results will pave the way for the development of future generations of sensors for hydrogen safety and detection.

## Figures and Tables

**Table 1:** Comparison of APTES-functionalized ZnO nanowires sensor performance with strategies derived from the literature, such as heterostructures, decoration with metal nanoparticles, and so on.

Material	Strategies	Hydrogen Concentration	Working Temperature	Response	Ref.
Ag/ZnO	hierarchical microstructures	50 ppm	250 °C	4.7	[46]
Pd-ZnO	nanosheets	50 ppm	250 °C	2.5	[34]
PdAu_alloy_@ZnO	core–shell nanoparticles	100 ppm	300 °C	80	[40]
Ag:Pd/ZnO	nanorods	100 ppm	275 °C	51.36	[47]
Ag/Pd-ZnO	nanoplates	500 ppm	400 °C	78	[48]
ZIF-8/Pd/ZnO	nanowires	50 ppm	200 °C	6.7	[42]
ZnO/SnO_2_	nanofibers	5 ppm	300 °C	90	[43]
PdO/ZnO	nanoflowers	100 ppm	350 °C	16	[49]
NiO/ZnO	heterostructures	1200 ppm	RT	0.7	[41]
Graphene/ZnO	nanocomposite	200 ppm	150 °C	3.5	[44]
Pd-doped rGO/ZnO-SnO_2_	nanocomposite	100 ppm	380 °C	9.4	[45]
rGO/SnO_2_/ZnO	nanocomposite	500 ppm	175 °C	18.3	[50]
APTES-ZnO	nanowires	50 ppm 200 ppm 500 ppm	200 °C	7.7 35.5 97.5	This work

## Data Availability

Data will be made available on reasonable request.

## Supplementary Materials

The following supporting information can be downloaded at: https://www.mdpi.com/article/10.3390/s24217011/s1. Figure S1. X-ray diffraction spectra of bare ZnO NWs. Figure S2. Response/recovery times of APTES functionalized ZnO NWs (Z_APTES) at 200 °C and 300 °C toward different concentration of H_2_. Figure S3. Comparison of response toward 500 ppm of H_2_ of bare and APTES (10 mM and 20 mM concentrations) functionalized ZnO NWs. Table S1. Detection limits, and values of constants A and B obtained by fitting the calibration curves with power law.
