# Supplementary material for: ZnO Nanowires/Self-Assembled Monolayer Mediated Selective Detection of Hydrogen
Source: Sensors (Basel). 2024 Oct 31;24(21):7011. doi: 10.3390/s24217011 (PMC11548179; doi:10.3390/s24217011)
Supplement: Supplementary file 1 [file sensors-24-07011-s001.zip › sensors-3242782-supplementary.pdf]

Supplementary Material

ZnO Nanowires/Self-assembled Monolayer Mediated Selective Detection of Hydrogen

Mandeep Singh[1], Navpreet Kaur[2]* and Elisabetta Comini[2]*

[1]Department of Physics, Politecnico Di Milano, Piazza Leonardo da Vinci 32, 20133 Milan, Italy

[2]SENSOR Laboratory, University of Brescia, Via D. Valotti 9, Brescia 25133, Italy A. B.

E-mail: mandeep.singh@polimi.it, navpreet.kaur@unibs.it

1. X-ray Diffraction Pattern of Bare ZnO Nanowires

Figure 1S report the GI-XRD of ZnO NWs grown on alumina substrate. The peaks labled as *, correspond to the alumina substrate. On the other hand, the observation of XRD peaks at 2θ=31.83°, 34.40°, 36.29°, 47.56°, and 56.70°, corresponds to the (100), (002), (101), (102) and (110) planes of ZnO NWs respectively.[1] The occurrence of these peaks confirms the polycrystalline nature of ZnO NWs with hexagonal wurtzite structure.[1,2]

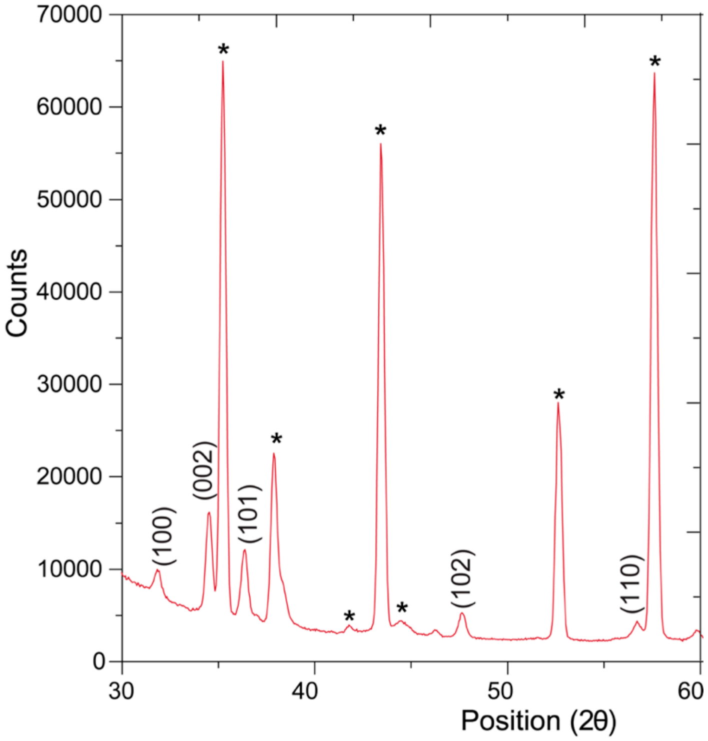

Figure S1. X-ray diffraction spectra of bare ZnO NWs

2. Response/Recovery Times of APTES Functionalized ZnO NWs Hydrogen Sensor

Figure S2 showed the response/recovely times of Z_APTES sensors at 200 °C and 300 °C toward the different concentration of H_2. It should be pointed out that both recovery/response times are overestimated because of the limitations of the test chamber which required 5-10 minutes to fill the full volume (1L) of stainless-steel chamber. Thus, in a real, much lesser values can be expected for both response and recovery times.

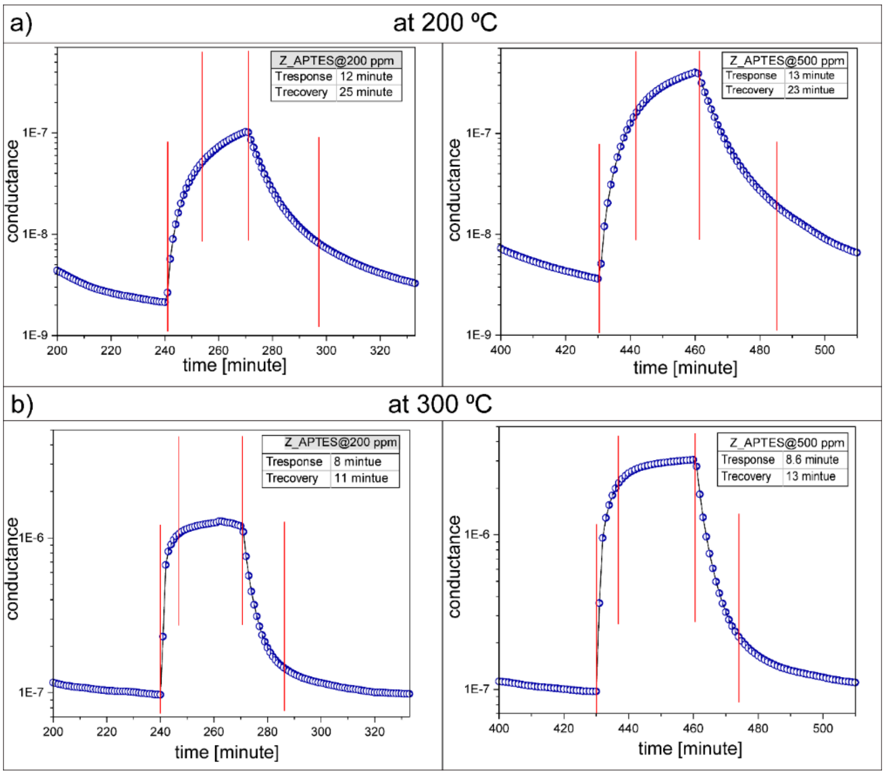

Figure S2. Response/recovery times of APTES functionalized ZnO NWs (Z_APTES) at 200 °C and 300 °C toward different concentration of H_2.

3. Detection Limits of Bare and APTES Functionalized ZnO Nanowires

Sensor	A	B	Detection limits
APTES-ZnO	0.1	1.1	7 ppm
ZnO	0.2	0.7	12 ppm

Table S1. Detection limits, and values of constants A and B obtained by fitting the calibration curves with power law.

4. Effect of Molar Ratio on the Sensing Performance

As it can be seen from the sensing data, the APTES functionalized ZnO NWs showed superior sensing performance as compared to bare ZnO NWs. Hence, we have increased the molar concentration of APTES to 20mM in an anticipation to further enhance the sensing performance. Particularly, we want to increase effect of negatively charged amine groups of APTES on the surface electron density of ZnO NWs. However, as it can be seen from figure 2S, instead of improvement, the sensing performance deteriorates. The reason behind this relies in the formation mechanism of APTES monolayer. The silanization of organosilanes on oxide surface requires a sufficient amount of water for the hydrolysis. However, both the deficiency and excess of water inside the SAM solution can harm the monolayer properties. As described in the experimental section of surface functionalization, APTES monolayer requires OH groups for their attachment on ZnO NWs. Specifically, APTES molecules immediately react with the OH groups on ZnO surface during the silanization via consuming adequate amount of water and forms the monolayer. As the reaction proceeds, the water molecules becomes resistant toward ZnO NWs surface and start reacting with the APTES molecules inside the solution. It has been observed that this effect is more dominating at higher concentrations of SAM solution. Hence, when the molar concentration of APTES was increased to 20mM, the reaction between APTES molecules and water inside the solution rather than reaction with the ZnO NWs surface, results into the occurrence of incomplete hydrolysis. This incomplete hydrolysis hinders the uniformity and reactivity of APTES monolayer. Hence, the incomplete hydrolysis of APTES monolayer at 20mM is the primary reason behind the inferior performance of functionalized sensor.

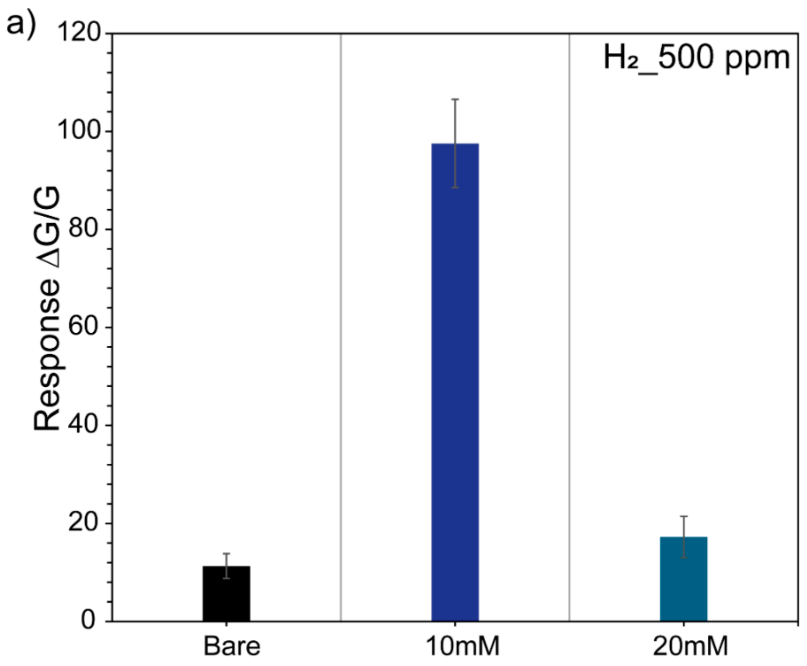

Figure S3. Comparison of response toward 500ppm of H2 of bare and APTES (10mM and 20mM concentrations) functionalized ZnO NWs.

References

[1] Singh, M.; Kaur, N.; Drera, G.; Casotto, A.; Ermenegildo, L.S.; Comini, E. SAM Functionalized ZnO Nanowires for Selective Acetone Detection: Optimized Surface Specific Interaction Using APTMS and GLYMO Monolayers. *Adv. Funct. Mater.* **2020**, *30*, 2003217.

[2] Lupan, O.; Emelchenko, G.A.; Ursaki, V.V.; Chai, G.; Redkin, A.N.; Gruzintsev, A.N.; Tiginyanu, I.M.; Chow, L.; Ono, L.K.; Roldan Cuenya, B.; et al. Synthesis and Characterization of ZnO Nanowires for Nanosensor Applications. *Mater. Res. Bull.* **2010**, *45*, 1026–1032.
